# Emerging biologic modalities for targeted protein degradation

**DOI:** 10.1016/j.jbc.2026.111248

**Published:** 2026-02-04

**Authors:** Alana G. Caldwell, Harshil Parmar, Xiaoyu Zhang

**Affiliations:** 1 Interdisciplinary Biological Sciences Graduate Program, Northwestern University, Evanston, Illinois, USA; 2 Department of Chemistry, Northwestern University, Evanston, Illinois, USA; 3 Chemistry of Life Processes Institute, Northwestern University, Evanston, Illinois, USA; 4 Robert H. Lurie Comprehensive Cancer Center, Northwestern University, Chicago, Illinois, USA; 5 Center for Human Immunobiology, Northwestern University, Chicago, Illinois, USA; 6 International Institute for Nanotechnology, Northwestern University, Evanston, Illinois, USA

**Keywords:** targeted protein degradation, biologic degraders, ubiquitin–proteasome system, E3 ubiquitin ligases, antibody and nanobody binders, peptide-based degraders

## Abstract

Targeted protein degradation (TPD) has emerged as a powerful approach for eliminating disease-associated proteins by harnessing the ubiquitin–proteasome system. Biologic degraders are modular protein chimeras that recruit ubiquitin machinery to target proteins. They offer high specificity, modular design, and the ability to access targets traditionally considered challenging for small-molecule ligands. This review surveys the expanding landscape of biologic TPD modalities, highlighting E3 ligase– and E2 enzyme–based degraders, TRIM-Away and TRIMbody-Away systems, and diverse biologics-based ligands that serve as target-binding components. We also discuss emerging peptide-based strategies, which bridge biologic and synthetic approaches. Finally, we highlight future opportunities to improve biologic degraders and their potential to expand the scope of TPD.

The ubiquitin–proteasome system is responsible for degrading approximately 80% of intracellular proteins and plays a critical role in maintaining proteostasis (1). In this pathway, a cascade of enzymes, including the E1-activating enzyme, E2-conjugating enzyme, and E3 ubiquitin ligase, mediates the activation and conjugation of ubiquitin to target proteins. Following ubiquitination, these proteins are directed to the proteasome, where they are cleaved into short peptides. E3 ubiquitin ligases confer substrate specificity either through dedicated substrate-binding proteins within multisubunit complexes or, in the case of single-unit E3 ligases, by directly recognizing and recruiting protein targets (2, 3). The growing field of intracellular targeted protein degradation (TPD) leverages this system through small molecules or protein-based approaches to induce the degradation of proteins of interest (POIs) (3, 4). An alternative degradation mechanism involves engaging cell surface receptors to direct POIs to the lysosome. This approach has been recently reviewed elsewhere and will not be discussed further in this review (5, 6).

TPD was first described by Crews and Deshaies in 2001 (7). In this seminal study, the authors synthesized a chimeric molecule composed of an IκBα phosphopeptide, recognized by the Skp1–Cullin–F box E3 ligase complex, and ovalicin, a methionine aminopeptidase-2 inhibitor. This molecule, dubbed “proteolysis-targeting chimera” (PROTAC), induced the degradation of methionine aminopeptidase-2 *via* the ubiquitin–proteasome system (7). This work demonstrated that induced proximity to an E3 ligase could effectively promote target protein degradation. Over the following 2 decades, PROTAC research shifted heavily toward small-molecule PROTACs (smPROTACs), which are heterobifunctional compounds composed of an E3 ligase–binding ligand, a POI-binding ligand, and a linker. smPROTACs targeting the androgen receptor and estrogen receptor entered clinical trials around 2019 as cancer therapeutics (3). Similarly, molecular glues, which are small molecules that stabilize weak or transient interactions between E3 ligases and their targets, have also been successful in promoting POI degradation (8, 9). Despite their promise, smPROTACs and molecular glues face several challenges, including the potential for off-target and toxic effects driven by polypharmacology, the need for both the E3 ligase and POI to be ligandable in the case of smPROTACs, and the lack of well-defined design principles for molecular glues (4). Alternatively, biologics-based heterobifunctional degraders are an emerging class of therapeutics that have the potential to offer high specificity, reduced off-target effects, and modular design (Fig. 1). This review highlights these emerging biologic modalities for TPD, with a focus on their design, challenges, and future opportunities.

**Figure 1:**
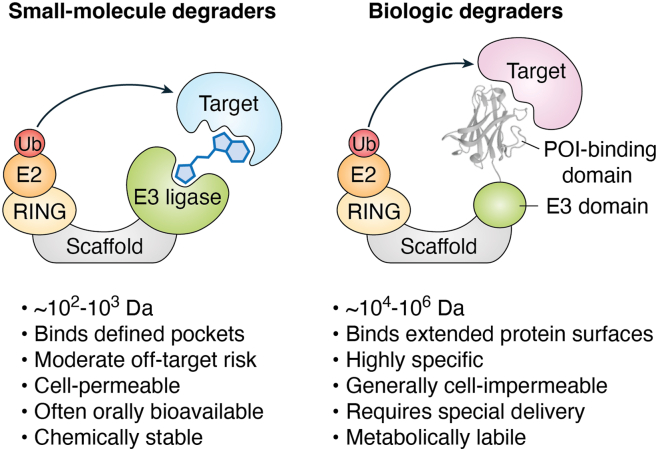
**Comparison of small-molecule and biologic degraders**. Overview of key distinctions between biologic-based and small-molecule degraders.

## Biologics-based heterobifunctional degraders

Biologics-based heterobifunctional degraders are modular protein chimeras designed to induce TPD. Like their small-molecule counterparts, biologic degraders harness the ubiquitin–proteasome system by recruiting E3 ligases to degrade POIs. A number of E3 ligases have been shown to support TPD, with smPROTACs commonly engaging cereblon and von Hippel–Lindau (VHL) (10). Nonetheless, a potential limitation of smPROTACs that engage these E3 ligases is the emergence of resistance because of genomic alterations or deletions that impair the endogenous E3 ligase machinery (11). Conversely, biologic degraders can introduce an exogenous E3 ligase or its functional domain, potentially bypassing this loss and overcoming acquired resistance.

Several formats of these biologic modalities have been developed, including ubiquibodies (12), chimeric F-box fusion proteins (13), affinity-directed protein missile (AdPROM) (14), antibody RING-mediated destruction (ARMeD) (15), and biological PROTACs (bioPROTACs) (16, 17) (Table 1). Although these strategies share the common principle of bringing a target protein into proximity with an engineered E3 ligase to trigger ubiquitination, they differ in their molecular design and choice of E3 machinery. Ubiquibodies and bioPROTACs use modular fusion of an E3 ligase or substrate adaptor (*e*.*g*., CHIP, SPOP, or RNF4) to an engineered target-binding domain, such as a nanobody or designed ankyrin repeat protein (DARPin) (12, 16, 17). F-box fusion proteins exploit the endogenous Skp1–Cullin–F box complex, in which the substrate-recognition domain of the F-box protein β-TrCP (also known as FBXW1) is replaced by a heterologous binder (*e*.*g*., nanobody or protein domain) (13). AdPROM uses an anti-GFP nanobody fused to the VHL adaptor, enabling degradation of GFP-tagged proteins (14), whereas ARMeD relies on the RING domain of RNF4 fused to a nanobody to mediate ubiquitination directly (15). These distinct architectures illustrate the diversity of biologic degrader designs and underscore how varying the E3 ligase component or target-binding module can tailor substrate scope, subcellular localization, and degradation efficiency.

**Table 1:** Summary of biologics-based degraders

Degrader modality	E3 ligases	Ligand types for POI	Construct size (kDa)	Degraded targets	Delivery method	Ref.
Ubiquibody/BioPROTAC	CHIP, VHL, NEDD4, SPOP, RNF4, βTrCP, FBW7, SKP2, CRBN (cereblon), SOCS2, and ASB1	Antibody, nanobody, monobody, and DARPin	Variable ∼30–200	GLB1, GFP fusions (*e*.*g*., H2B, KRAS, PCNA, SHP2), BCL11A, KRAS, PCNA, SHP2, RAS GTPases, ACTN, EGFR, and SNCA	Transfection, extracellular incubation, lipid nanoparticle delivery, and microinjection	(12, 16, 17, 18, 19, 22, 23, 24, 28, 29)
F-box fusion protein	F-box domain	Protein domain, nanobody	Variable ∼40–65	CTNNB1, fluorescent protein fusions (*e*.*g*., H2B, His2Av, Crb, Pck-TEVpcs, α-Cat), and RHO GTPases	Transfection, lentiviral transduction, and microinjection	(13, 26, 27)
AdPROM	VHL	Anti-GFP nanobody	∼97	GFP fusions (*e*.*g*., VPS34, PAWS1)	Retroviral transduction	(14)
ARMeD	RING domain of RNF4 (single domain or dimer)	Nanobody	∼25–30	Fluorescent protein fusions (*e*.*g*., PARG, PEX10, RNF146, PML, SP100, TUBA1A), NEDP1	Transfection, electroporation, and microinjection	(15)
TRIM-Away	TRIM21	Antibody	∼200	Cytosolic, membrane-anchored and nuclear GFP, GFP fusions (*e*.*g*., H2B), Eg5, Rec8, disease-variant HTT, ERK1, IKKA, PCNT, MTOR, NFKBIA, NLRP3, NUP98, NUP214, NUP62, CENPA, Ddx19b, Dicer1, Mitfa, Msrb3, HER2, HER3, STAT3, Lbh, PACSIN2, and BCL11A	Electroporation, microinjection	(17, 33, 36, 37, 38, 40)
TRIMbody-Away	TRIM21	Nanobody	∼40–70	EGFP, MALT1, EED, HuR, BIRC5, and ASFV-encoded proteins (p30, p54, p72)	Transfection, lentiviral transduction, and electroporation	(20, 21, 34, 35, 41)

## E3 ubiquitin ligase for biologic degraders

**Figure 2:**
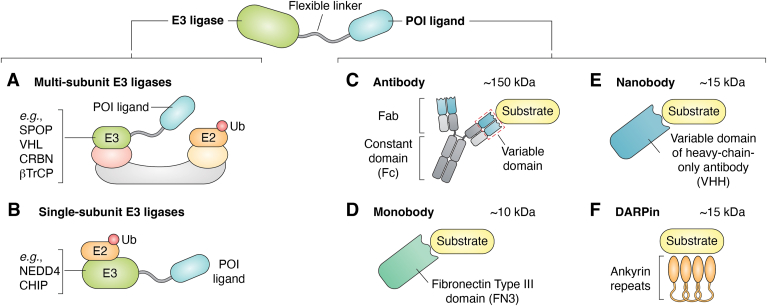
**Overview of biologic degrader designs and strategies**. *A*, multisubunit E3 ligases. Full-length or truncated multicomponent E3 ligases (*e*.*g*., SPOP, von Hippel–Lindau [VHL], CRBN, β-TrCP) can be fused to a target-binding ligand to recruit neosubstrates for degradation. *B*, single-subunit E3 ligases. E3 ligases such as NEDD4 or CHIP can be used in biologic degraders, in which truncated variants retain catalytic activity, whereas their substrate-recognition domains are replaced with target-binding modules. *C*, antibody ligands. Multidomain protein complexes that recognize specific antigens *via* their variable heavy and light chains. *D*, monobody ligands. Synthetic single-domain β-sandwich proteins derived from the fibronectin type III (FN3) domain, engineered for high affinity and specificity through loop diversification. *E*, nanobody ligands. Camelid-derived single-variable heavy-chain domains capable of binding deep or cryptic surface pockets on target proteins. *F*, designed ankyrin repeat proteins (DARPins). Human-derived, single-domain scaffolds composed of stacked ankyrin motifs that can be engineered for high-affinity or multispecific binding. CRBN, cereblon.

To date, multiple E3 ligase families, including Cullin-RING ligases (*e*.*g*., SPOP), single-subunit RING ligases (*e*.*g*., tripartite motif [TRIM]21), HECT ligases (*e*.*g*., NEDD4), and U-box ligases (*e*.*g*., CHIP), have been shown to function effectively in biologic degrader constructs (12, 16, 18, 19, 20, 21, 22, 23, 24) (Fig. 2, *A* and *B*). The Partridge group used a system in which GFP was fused to histone 2B or KRAS and demonstrated that an anti-GFP nanobody (vhhGFP4) fused to five different E3 ligases, including FBXW1, SKP2, VHL, SPOP, and CHIP, achieved over 70% degradation of cellular GFP (16, 18). Moreover, both prokaryotic and nonhuman eukaryotic E3 ligases have been incorporated into biologic degrader designs, underscoring the versatility of this approach (25, 26). For example, the bacterial E3 ligase, IpaH9.8, when fused to various monobodies, induced potent degradation of fluorescently tagged mammalian proteins, including histone 2B, SHP2, and EGFR (25). Similarly, the *Drosophila melanogaster* F-box E3 ligase Slmb, when fused to nanobodies (NSlmb-vhhGFP4 or SlmbF-box), robustly degraded targets in fly embryos and human tumor cell lines (26, 27). The flexibility in the choice of E3 ligase allows these constructs to be applied in a wide array of biological systems.

A key advantage of the diverse range of E3 ligases capable of mediating target degradation *via* biologic degraders is their ability to tailor constructs to specific cellular contexts and targets, such as exploiting the endogenous subcellular localization of E3 ligases to promote degradation within specific compartments. The Cullin-RING ligase substrate adaptor SPOP, for instance, is predominantly nuclear and has been harnessed in bioPROTAC systems to degrade nuclear proteins, such as proliferating cell nuclear antigen, thereby suppressing tumor growth (16, 23, 28, 29). Furthermore, E3 ligases or their functional domains used in biologic degraders can be redirected to desired organelles by incorporating localization sequences (23). Continued development of biologic degraders that harness these E3 ligases offers a promising strategy for achieving highly specific degradation in subcellular environments that often present challenges for small-molecule degraders.

## Exploiting TRIM E3 ligases for the development of biologic degraders

**Figure 3:**
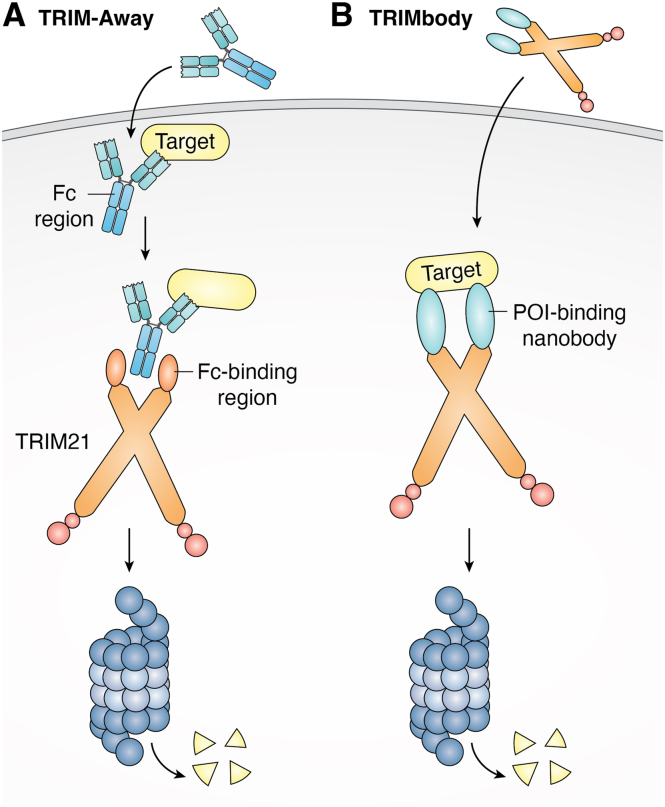
**TRIM-Away and TRIMbody-Away strategies for targeted protein degradation**. *A*, TRIM-Away mechanism. TRIM21 recognizes the Fc domain of antibodies and directs degradation of both the antibody and its bound substrate. Exogenous introduction of antibodies against neosubstrates can induce TRIM21-dependent target degradation, a strategy termed “TRIM-Away.” *B*, TRIMbody-Away mechanism. Cells with low TRIM21 expression can be transfected with engineered TRIM21 variants wherein the Fc recognition domains are replaced with target-binding ligands (TRIMbodies) to direct target degradation. TRIM, tripartite motif.

TRIM E3 ligases comprise a family of approximately 80 proteins in humans that share a conserved RING domain (30). TRIM21 is unique for its high affinity toward the Fc domain of immunoglobulins (31). Endogenously, TRIM21 recognizes antibody-bound pathogens during infection (32). TRIM-Away is a method that exploits this pathway for TPD applications (Fig. 3*A*, Table 1). In the initial study, Clift *et al*. (33) microinjected or electroporated immunoglobulin G against nine targets and observed TRIM21-mediated degradation, achieving near-complete elimination. Notably, TRIM21 is expressed at relatively high endogenous levels, which are sufficient to support efficient degradation and enable the TRIM-Away system to function robustly across different cellular contexts. TRIM-Away is also active in multiple subcellular locations, primarily in the cytosol, and can target proteins associated with internalized vesicles or the plasma membrane following endocytosis. Moreover, by fusing an Fc domain to a nanobody (33), TRIM-Away constructs can be made smaller and used to target nuclear-localized proteins that are otherwise difficult for full-size immunoglobulin G molecules to access.

To further reduce the size of biologic constructs used for TRIM-Away, Chen *et al*. (20) described a modified strategy termed TRIMbody-Away, in which a truncated TRIM21 lacking the Fc recognition domain is directly fused to nanobodies targeting a POI (Fig. 3*B*, Table 1). Using this approach, they successfully induced degradation of intracellular enhanced GFP. Notably, treatment with either a proteasome inhibitor or an autophagy-lysosome inhibitor suppressed degradation, indicating that both proteasome- and lysosome-mediated pathways are potentially involved. While TRIM-Away requires only antibody or nanobody microinjection in cells with sufficient endogenous TRIM21, cells with low TRIM21 expression need to receive both the ligase and the antibody to achieve efficient target degradation. TRIMbody-Away addresses this by combining the target-recruiting domain and ligase into a single fusion, which may improve uptake efficiency and reduce the risk of interfering with the native functions of TRIM21. Both TRIM-Away and TRIMbody-Away represent versatile technologies for biological research and potential therapeutic applications (34, 35, 36, 37, 38, 39).

In a recent study investigating the therapeutic effects of TRIM-Away–mediated protein degradation in breast cancer, Dey *et al*. (40) employed electroporation and antibody transfection to degrade HER2, HER3, and STAT3. As a result, degradation of these targets inhibited downstream signaling pathways for up to 8 days. This work demonstrated that direct removal of oncogenic proteins can reduce tumor growth without activating compensatory pathways. Moreover, this work demonstrated that TRIM-Away can target specific post-translational modifications, offering an additional layer of specificity and expanding the scope of this technology. For instance, phospho-activated forms of STAT3 were selectively degraded without affecting the total protein pool.

## E2 enzymes for biologic degraders

E2 ubiquitin–conjugating enzyme-based biologic degraders function similarly to E3 ligase–based degraders by engaging an upstream component of the ubiquitin–proteasome system. Work by Hunt and Taylor (42) demonstrated that chimeric E2 enzymes, including UBE2B and UBE2D1, could recognize and degrade targets, such as SHP2 and KRAS. This complementary approach has the potential to recognize a wide range of protein substrates and promote their degradation, owning to the promiscuity and abundance of E2 enzymes. Future research on E2-based biologic degraders should aim to identify additional E2 enzymes capable of promoting TPD and to assess whether they possess broader or distinct substrate specificities compared with UBE2B and UBE2D1. Another important direction is the development of methods to tune the activity of these E2 enzymes, enabling target degradation without disrupting their native functions or regulated degradation pathways. Overall, E2-based biologic degraders represent a promising new avenue for expanding TPD beyond traditional E3 ligase–centric approaches.

## Target-binding ligands for biologic degraders

Small-molecule degraders generally require highly specific chemical ligands that bind to a surface pocket of the target protein. Many proteins, however, lack suitable pockets for developing high-affinity chemical ligands, creating a significant barrier to targeting a broader range of proteins. As a result, the scope of small-molecule degraders is largely limited to conventionally druggable proteins, such as enzymes and receptors, which constitute only about 15% of the proteome (43). One advantage of biologic degraders over small-molecule degraders is the wide variety of target-recruiting elements that can be employed in their design. Biologic-based ligands, such as antibodies, monobodies, and DARPins, bind target proteins through one or more complementary peptide sequences (Fig. 2, *C*–*F*), thereby providing high-affinity ligands for challenging targets that are often considered chemically inaccessible. To identify these ligands, display technologies, such as phage display and yeast display, enable the selection of peptide or protein binders to POIs from large combinatorial libraries (44). Moreover, these biologic ligands can be selected or further tuned to minimize crossreactivity with homologous proteins and isoforms or to act as broad recruiters of a protein family.

KRAS is a well-studied oncogene with a high mutation rate in a variety of cancers and is associated with poor prognosis (45). Historically, KRAS has been difficult to target with small molecules. Although recent advances in small molecule development have partially overcome this limitation, these efforts remain largely restricted to specific KRAS mutations, such as G12C (45) and G12D (46). In contrast, biologic ligands that can target a broader panel of KRAS mutants have the potential to selectively engage oncogenic KRAS in cancer cells while sparing normal cells expressing wildtype KRAS. Among such biologic ligands, monobodies represent a promising class. These single-domain antibody fragments (∼10–15 kDa) are substantially smaller than conventional antibodies (∼150 kDa) (47) (Fig. 2, *C* and *D*). Their reduced size facilitates efficient transport between cellular compartments and allows for scalable production and optimization *via* directed evolution platforms (47). In a recent work, a screen for monobodies against KRAS identified a construct that selectively binds the G12V and G12C mutants without engaging wildtype KRAS (48). Beyond mutation selectivity, monobodies also have the potential to differentiate between homologous proteins and specific conformational states. For example, RHO GTPases switch between active GTP-bound and inactive GDP-bound states, and RHOA, RHOB, and RHOC share 85% sequence conservation (49). Work by Bery *et al*. identified a monobody that selectively recognizes active GTP–bound RHOB but not GDP-bound RHOB or either RHOA or RHOC (27). When fused to the F-box domain of Slmb, a component of the F-box E3 ligase complex in Drosophila (26), this monobody effectively inhibits GTPase signaling by inducing proteasomal degradation of the GTP-bound RHOB (27). This approach demonstrates a promising strategy for selectively removing the active forms of state-switching proteins.

Nanobodies are camelid-derived single-domain antibodies (∼15 kDa), also known as variable domains of heavy chain–only antibodies (VHHs) (Fig. 2*E*). Owing to their high stability, small size, and ease of production, nanobodies have rapidly emerged as a versatile platform for both research and therapeutic applications (50). Several nanobody-based degrader systems have been developed. In the ARMeD approach, a nanobody is fused to the RING domain of RNF4 to recruit and degrade specific targets (15). Similarly, the AdPROM system employs an anti-GFP nanobody fused to VHL to direct the degradation of GFP-tagged proteins (14). The rapidly expanding repertoire of nanobodies that recognize disease-relevant targets highlights their transformative potential for developing selective and programmable biologic degraders.

DARPins are small (∼15 kDa), single-domain proteins that can be selected to bind targets with high specificity and affinity (51) (Fig. 2*F*). Bery *et al*. (52) screened for anti-KRAS DARPins and identified two, K13 and K19, which selectively bound and inhibited KRAS mutants in the colorectal cancer cell line HCT116. Although these DARPins could bind wildtype KRAS, they did not inhibit its function (52). In a follow-up study, the authors used the KRAS-specific DARPin K19 to generate a KRAS mutant degrader (24). Notably, comparison with pan-RAS degraders revealed that the KRAS-specific degrader selectively inhibited the proliferation of tumors harboring mutant KRAS without affecting cells expressing wildtype KRAS, whereas the pan-RAS degraders suppressed proliferation of all cell types tested. Collectively, these studies highlight that the nature of the target ligand critically influences the specificity and breadth of biologic degraders toward their intended POI.

## Peptide-based degraders: strategies and emerging modalities

**Figure 4:**
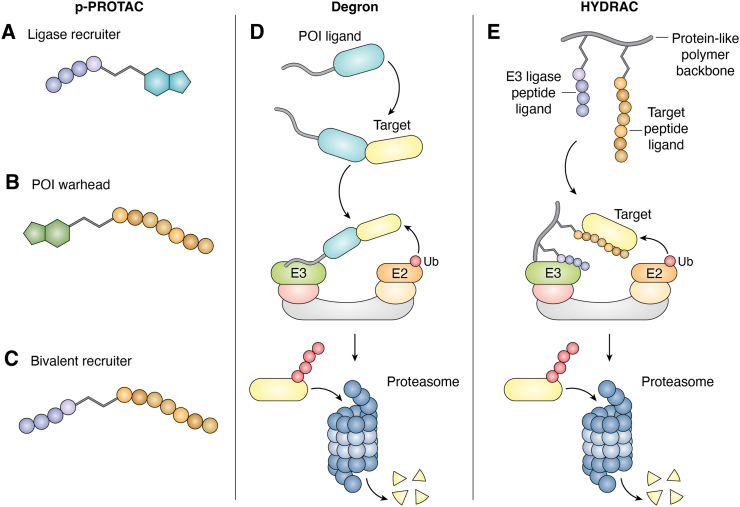
**Peptide-based biologic degraders**. *A*, peptide as E3 ligase recruiter. A peptide ligand is used to recruit the E3 ligase in the design of peptide-based PROTACs (p-PROTACs). *B*, peptide as POI recruiter. A peptide ligand derived from a target-binding motif engages the POI to promote its ubiquitination. *C*, peptide as bivalent recruiter. Two distinct peptide ligands are linked to simultaneously engage both the E3 ligase and the POI, eliminating the need for small-molecule components. *D*, degron-based degraders. A degron sequence fused to a target ligand is recognized by an E3 ligase upon POI binding, leading to ubiquitination and proteasomal degradation. *E*, HYDRACs (HYbrid DegRAding Copolymer). Polymer-linked assemblies of multiple peptide ligands for the E3 ligase and POI promote multivalent engagement and efficient target degradation. POI, protein of interest; PROTAC, proteolysis-targeting chimera.

Peptide-based degraders use peptides to engage E3 ligases, target proteins, or both, promoting POI degradation *via* induced proximity to the ubiquitination machinery. Depending on their design, peptides can serve distinct functional roles. In one format, peptides act as E3 ligase recruiters by mimicking endogenous degron motifs or E3-binding sequences to recruit the ligase (Fig. 4*A*). Alternatively, peptides can function as POI warheads, binding directly to the target protein to facilitate its ubiquitination when coupled to a suitable E3-binding module (Fig. 4*B*). A third strategy employs bivalent peptide recruiters that incorporate two distinct peptide ligands, one for the E3 ligase and one for the POI, thereby eliminating the need for small-molecule components (Fig. 4*C*). Peptide-based binders can offer advantages over fully synthetic small molecules, particularly for proteins with shallow pockets that are challenging to target with small-molecule ligands. Owing to their larger surface engagement, peptides can interact with a broader range of E3 ligases and targets, thereby expanding the scope of degradable proteins. Studies have shown that their high specificity and low toxicity enable peptide-based degraders to induce potent degradation of oncogenic proteins, thereby inhibiting cell growth, migration, and invasion (53, 54, 55, 56, 57). Moreover, recent advances in the computational design of peptide-based binders for POIs, such as PepMLM, have the potential to further expand the druggable space of peptide-based degraders and have been demonstrated to be effective in degrader development (58).

Another class of peptide-based degraders is based on degron-tagged POI ligands. Degrons are short peptide sequences recognized by E3 ligases that promote protein degradation (59). By fusing degrons to biologic ligands for specific targets, these degron-mediated pathways can be harnessed for efficient target degradation (Fig. 4*D*). For example, degron sequences can recruit ubiquitin ligases *via* an N-terminal arginine residue or a C-terminal PEST sequence (rich in proline, glutamic acid, serine, and threonine) (60, 61). Work by Tamaki *et al*. (62) demonstrated that a single-chain variable fragment targeting TAR DNA–binding protein 43 aggregates, a key driver of amyotrophic lateral sclerosis, fused to a PEST-like sequence, effectively eliminated TAR DNA–binding protein 43 aggregation. Similarly, fusing degron sequences to ligands of misfolded Huntingtin protein or α-synuclein allowed clearance of protein aggregates, showing promising applications for Huntington’s and Parkinson’s diseases, respectively (63, 64, 65). Notably, studies have shown that adding a membrane-permeating sequence can efficiently promote cellular uptake of peptide-based degraders (63). Recently, Kim *et al*. engineered degron-based degraders to control chimeric antigen receptor (CAR)-T cell signaling (66). Because excessive CAR signaling can lead to severe inflammation and T-cell exhaustion (67), they designed an environmentally responsive circuit that induces degron-mediated degradation of the essential T-cell kinase Zap70, thereby suppressing CAR-T cell activity (66). Together, these studies highlight the broad potential of degron-tagged ligands as modular platforms for targeted protein elimination, with significant potential for future therapeutic development.

Recently, a new peptide-based modality, termed HYbrid DegRAding Copolymer (HYDRAC), was reported to utilize polymer-conjugated peptides to promote TPD (68) (Fig. 4*E*). By linking multiple peptide units through a polymer backbone, HYDRAC enhances binding avidity and stability compared with conventional peptide–based degraders. This approach has been shown to degrade the transcription factor MYC and suppress tumor growth, demonstrating both effective target engagement and therapeutic potential. In addition, HYDRAC addresses common limitations of traditional peptides (*e*.*g*., low biological stability and poor cell permeability) through its hydrophobic norbornenyl polymer backbone, which shields peptide segments from proteolysis and promotes cellular uptake *via* increased hydrophobic interactions and endocytic internalization. This design thereby expands the potential applicability of peptide-based degraders.

## Limitations of biologic degraders

Despite significant advances in biologics-based degrader technologies, several limitations remain to be addressed. A primary challenge is their poor cell permeability, which restricts their cellular uptake and limits *in vivo* efficacy. Many biologic degraders are large, multidomain protein constructs with limited ability to penetrate the plasma membrane. Consequently, alternative delivery strategies, such as lipid nanoparticle encapsulation or viral vector delivery, are typically required. Of promise, Shen *et al*. developed a cell-permeable bioPROTAC composed of an anti-BCL11A nanobody, a cell-penetrating miniature protein (ZF5.3) engineered with a cationic and amphipathic surface to facilitate membrane translocation, and a truncated E3 ligase (SPOP or RNF4). This construct accumulated in the nucleus following extracellular incubation and reduced BCL11A levels by 70% after 12 h (17). This study highlights that limited cell permeability of biologic degraders can be overcome through protein engineering and/or delivery optimization.

Another important consideration for biologics-based degraders is their potential immunogenicity. The use of nonhuman scaffolds, such as camelid-derived nanobodies, bacterial ligases, or *Drosophila* F-box domains, may increase the likelihood of antidrug antibody formation upon repeated administration. Even human or humanized proteins can expose novel epitopes when fused to engineered domains or linkers. Strategies to mitigate these risks include deimmunization of nonhuman sequences through epitope prediction and mutagenesis, the incorporation of human-derived or humanized scaffolds, and careful evaluation of immune responses during preclinical development. Establishing low-immunogenicity designs will therefore be crucial for advancing biologic degraders toward clinical translation.

Beyond cellular uptake and immunogenicity, additional challenges include achieving tissue-specific distribution, minimizing off-target degradation, and ensuring sufficient expression of the recruited E3 ligase within the target cell type. These issues can be addressed through careful selection of ligases with compatible subcellular localization and expression profiles, the design of high-affinity and/or isoform-specific target ligands, and the use of delivery systems engineered for tissue- or cell-type specificity. Such design considerations will be essential for translating biologic degraders into clinically effective therapeutics.

## Summary and outlook

TPD has rapidly evolved from its conceptual origins in the early 2000s to an expanding set of therapeutic modalities. smPROTACs and molecular glue degraders have demonstrated the feasibility of redirecting the ubiquitin–proteasome system for protein elimination and are progressing in clinical trials. Nevertheless, these approaches face challenges, including dependence on ligandable surfaces, the emergence of resistance mechanisms, and limited design principles. Biologic degraders have emerged as a complementary class of TPD strategies that overcome some of these barriers by expanding the repertoire of ligases and ligands that can be harnessed for targeted degradation. Leveraging diverse E3 ubiquitin ligases, E2-conjugating enzymes, and peptide- or antibody-based binders, biologic degraders provide modularity, high specificity, and the ability to target proteins traditionally considered inaccessible. Recent work highlights their potential in addressing challenging oncogenic proteins, such as KRAS mutants, in selectively targeting conformational or specific states, and in developing versatile degrader platforms such as TRIM-Away and TRIMbody-Away. Together, these advances underscore the unique promise of biologics in expanding the landscape of TPD.

Looking forward, recombinant protein technologies are poised to push the boundaries of proteome manipulation, with emerging strategies offering both degradative and stabilizing capabilities. For example, novel constructs, such as ligand-ubiquitin fusion proteins, may overcome orientation and engagement limitations inherent to degrader design (69), whereas biologic DUBTACs could stabilize proteins by removing ubiquitin chains, offering a direct counterpoint to degradation (70, 71, 72, 73). Beyond proteasome-mediated degradation, lysosomal- and autophagy-based strategies, together with induced proximity platforms for other post-translational modifications (such as phosphorylation-targeting chimeras, acetylation-targeting chimeras, and phosphorylation-inducing chimeras) (74), will further expand the scope of proteome editing to include extracellular proteins, aggregates, organelles, and nucleic acids.

The next phase of biologic degraders will require both technical advances and conceptual innovation: improving delivery systems for large biologics, optimizing degrader formats for specific subcellular environments, and integrating strategies to minimize off-target effects. Equally important will be translating these tools into clinically viable therapeutics and demonstration of efficacy in complex disease models. Biologic degraders, by virtue of their modularity and specificity, are positioned to diversify therapeutic strategies and expand our ability to reprogram proteostasis. As the field advances, these modalities are expected to play a crucial role in both fundamental biology and next-generation precision medicines.

## Conflict of interest

The authors declare that they have no conflicts of interest with the contents of this article.
